# Immune evasion in esophageal squamous cell cancer: From the perspective of tumor microenvironment

**DOI:** 10.3389/fonc.2022.1096717

**Published:** 2023-01-09

**Authors:** Rongyang Li, Bing Huang, Hui Tian, Zhenguo Sun

**Affiliations:** Department of Thoracic Surgery, Qilu Hospital of Shandong University, Jinan, Shandong, China

**Keywords:** esophageal squamous cell cancer, immune evasion, tumor microenvironment, immunosuppression, immunotherapy

## Abstract

Esophageal cancer (EC) is one of the most life-threatening malignancies worldwide. Esophageal squamous cell carcinoma (ESCC) is the dominant subtype, accounting for approximately 90% of new incident EC each year. Although multidisciplinary treatment strategies have advanced rapidly, patients with ESCC are often diagnosed at advanced stage and the long-term prognosis remains unsatisfactory. In recent decades, immunotherapy, such as immune checkpoint inhibitors (ICIs), tumor vaccines, and chimeric antigen receptor T-cell (CAR-T) therapy, has been successfully used in clinical practice as a novel therapy for treating tumors, bringing new hope to ESCC patients. However, only a small fraction of patients achieved clinical benefits due to primary or acquired resistance. Immune evasion plays a pivotal role in the initiation and progression of ESCC. Therefore, a thorough understanding of the mechanisms by which ESCC cells escape from anti-tumor immunity is necessary for a more effective multidisciplinary treatment strategy. It has been widely recognized that immune evasion is closely associated with the crosstalk between tumor cells and the tumor microenvironment (TME). TME is a dynamic complex and comprehensive system including not only cellular components but also non-cellular components, which influence hallmarks and fates of tumor cells from the outside. Novel immunotherapy targeting tumor-favorable TME represents a promising strategy to achieve better therapeutic responses for patients with ESCC. In this review, we provide an overview of immune evasion in ESCC, mainly focusing on the molecular mechanisms that underlie the role of TME in immune evasion of ESCC. In addition, we also discuss the challenges and opportunities of precision therapy for ESCC by targeting TME.

## 1 Introduction

Esophageal cancer (EC) is an aggressive malignancy and accounts for the majority of cancer-related deaths worldwide, ranking seventh in incidence and sixth in mortality currently (1, 2). Generally, EC is divided into two subtypes based on pathological and biological features: esophageal squamous cell carcinoma (ESCC) and esophageal adenocarcinoma (EAC) (3). ESCC is the most common and the dominant subtype in Asians, accounting for approximately 90% of new incident EC each year, while EAC is more commonly diagnosed in western countries (4). Despite the great improvement in earlier diagnosis and multidisciplinary cancer treatment (including surgery, radiotherapy, chemotherapy and targeted therapy), the long-term prognosis of patients with ESCC remains unsatisfied (5), and the burden caused by ESCC continues to increase with population growth and aging (6). In recent decades, immunotherapy has gradually attracted attention due to the progressive insights into tumor immunology. Immunotherapy is a series of treatments aimed at revitalizing the anti-tumor immune system, restarting and sustaining the cancer-immune cycle, ultimately causing the death of tumor cells (7). Immune checkpoint inhibitors (ICIs), as a representative of immunotherapy, has been successfully used in the clinical practice for the treatment of ESCC with satisfactory outcomes (8, 9). However, only a small fraction of patients with ESCC response to immunotherapy due to primary or acquired resistance (10). Tumor cells can escape the immune system’s attack in a variety of ways such as inhibition of immune cells and restriction of antigen recognition, which is defined as immune evasion (11). Therefore, a thorough understanding of the mechanisms of immune evasion within the tumor microenvironment (TME) is necessary for a more effective multidisciplinary treatment strategy.

TME is a dynamic complex and comprehensive system, which includes not only cellular components such as tumor cells themselves and inflammatory/immune cells, but also non-cellular components such as extracellular matrix (ECM) and cytokines (12). During the past few decades, accumulative evidence has proven that TME plays a vital role in tumorigenesis, progression and immune escape of human cancer (13). To overcome the limitations of immunotherapy and develop more effective therapeutic strategies for ESCC, therapeutic strategies targeting tumor cells and tumor-favorable microenvironment emerged as a promising approach (14). In this review, we provide an overview of immune evasion in ESCC, mainly focusing on the molecular mechanisms that underlie the role of TME in immune evasion, especially in ESCC. In addition, we also discuss the challenges and opportunities of precision therapy for ESCC by targeting tumor-favorable TME.

## 2 Cellular components

### 2.1 ESCC cells

**Figure 1:**
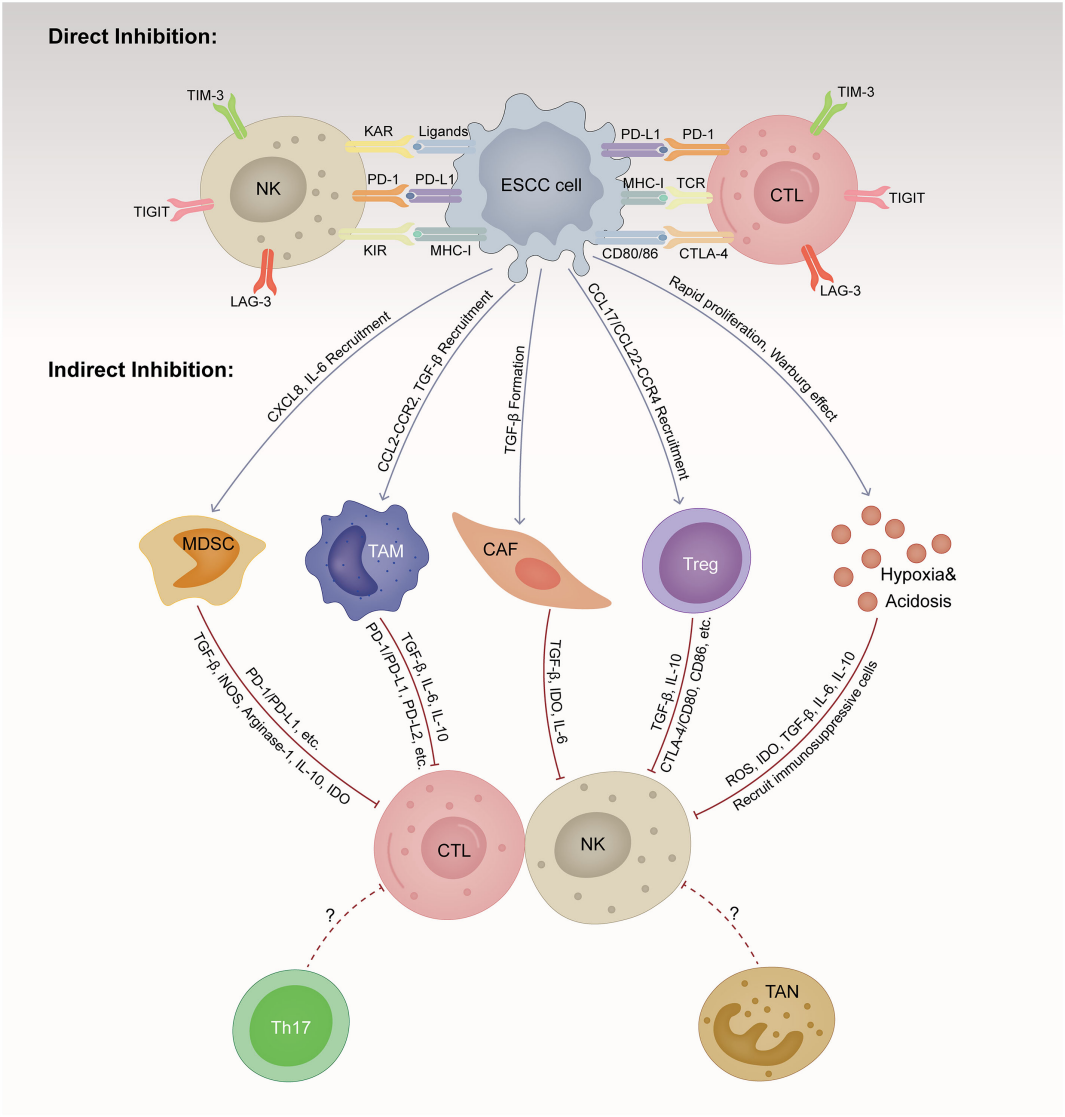
Immunosuppressive tumor microenvironment (TME) in esophageal squamous cell cancer (ESCC). TME is a dynamic complex and comprehensive system, composed of various cellular components and non-cellular components. ESCC cells are born to create an immunosuppressive milieu suppressing the proliferation and function of cytolytic NK and T cells directly and indirectly. ESCC, esophageal squamous cell cancer; CTL, cytotoxic T lymphocyte; NK, natural killer cell; MDSC, myeloid-derived suppressor cell; TAM, tumor-associated macrophage; CAF, cancer-associated fibroblast; Treg, regulator T cell; TAN, tumor-associated neutrophils; Th17, T helper cell 17; MHC-1, major histocompatibility class I; TCR, T cell receptor; KAR, killer activation receptor; KIR, killer inhibitory receptor; PD-1, programmed cell death protein 1; PD-L1, programmed cell death ligand 1; PD-L2, programmed cell death ligand 2; CTLA-4, cytotoxic T lymphocyte-associated antigen 4; LAG-3, lymphocyte activation gene-3; TIM-3, T-cell immunoglobulin and mucin domain-containing protein-3; TIGIT, T-cell immunoglobulin and immunoreceptor tyrosine-based inhibitory motif domain; TGF-β, transforming growth factor-β; IL, interleukin; CXCL, CXC motif chemokine ligand; CCL, CC motif chemokine ligand; CCR, CC motif chemokine receptor; IDO, indoleamine 2,3-dioxygenase; iNOS, inducible nitric oxide synthase-2; ROS, reactive oxygen species.

To effectively eradicate ESCC cells, repeated and magnified events of the cancer-immune cycle are indispensable, among which the initiation of identifying and processing tumor antigens recognition and ultimately the elimination of cancer cells taking place in the TME (15). However, ESCC cells are born to create an immunosuppressive milieu that suppresses the proliferation and function of cytolytic NK and T cells directly and indirectly (16). Here, we summarize the main mechanisms by which ESCC cells induce immune evasion (**Figure 1**).

Upregulation of immune checkpoint molecules is the main mechanism by which ESCC cells escape attacks from the immune system. Immune checkpoints (ICs) are immunosuppressive molecules that are normally expressed on the surface of multiple immune cells, which play an important role in preventing the occurrence of autoimmunity and long-lasting inflammation (17). However, ESCC cells can highly express the ligands for ICs to protect themselves from being killed by immune cells. At present, programmed cell death protein 1 (PD-1) and programmed cell death ligand 1 (PD-L1) are the most well-studied ICs in ESCC. Clinicians usually use the tumor proportion score (TPS) and combined positive score (CPS) to assess the expression of PD-L1, which is calculated by the following immunohistochemical scoring algorithm: TPS = (total number of PD-L1 stained tumor cells/total number of tumor cells) × 100%; CPS = (total number of PD-L1 stained cells/total number of tumor cells) × 100. It has been reported that patients with ESCC with TPS greater than 10% or CPS greater than 10 could benefit more from PD-1 inhibitors, which typically account for 30-50% of patients with ESCC (18). Abnormally highly expressed PD-L1 on tumor cells can bind to PD-1 expressed on various immune cells, protecting tumor cells from being lysis by CD8^+^ T cells or NK cells (19). Moreover, the binding of PD-1 and PD-L1 could also activate intercellular signaling pathways, inhibiting the function and proliferation of effector T cells (20). Tumor cells can even suppress T-cell immunity by secreting PD-L1 through exosomes (21). Antibody-based immunotherapy that blocks the interaction between PD-1 and PD-L1 has achieved favorable therapeutic effects for the treatment of ESCC (22). Cytotoxic T lymphocyte-associated antigen 4 (CTLA-4) is another well-recognized IC, which is usually expressed on the surface of regulator T cells (Tregs) and T lymphocytes. CTLA-4 negatively regulate the anti-tumor immunity of T cells by binding to B7 ligand family. It has been widely demonstrated that the overexpression of CTLA-4 is associated with reduced interleukin-2 (IL-2) expression and T-cell cycle arrest, resulting in the reduction of T-cell function and immune evasion of cancer cells (23). A phase II clinical trial has shown the CTLA-4 inhibitor tremelimumab could provide favorable survival benefits for patients with gastric cancer and EAC (24). The clinical trial CheckMate-648 has proved that ipilimumab (anti-CTLA-4) combined with nivolumab (anti-PD-1) in first-line treatment could improve overall survival (OS) with durable objective responses and acceptable safety compared to chemotherapy alone for ESCC patients (25). However, due to the limited number of available clinical trials, the efficiency and safety of CTLA-4 inhibitors as an emerging immunotherapy for ESCC needs to be further investigated. Newly emerging ICs, including lymphocyte activation gene-3 (LAG-3), CD47, T-cell immunoglobulin and mucin domain-containing protein-3 (TIM-3), and T-cell immunoglobulin and immunoreceptor tyrosine-based inhibitory motif domain (TIGIT), have been identified to date and their efficacy is being investigated in various stages of preclinical and clinical trials. Previous studies have demonstrated their remarkable immune inhibitory effects on lymphocytes in ESCC and elevated expression of these molecules in ESCC patients was associated with worse survival outcomes, indicating that these ICs are promising targets for immunotherapy of ESCC (26–29). However, identification of a reliable immunotherapy that targets a certain IC remains a severe challenge because of the high heterogeneity of ICs expression on the cancer cell surface (30).

In addition to upregulating ICs, the expression of neoantigens and major histocompatibility class (MHC) I molecules on tumor cells is usually downregulated or completely absent, preventing the recognition of activated T cells and NK cells (11). Downregulation and depletion of neoantigens have been identified among various malignancies, such as lung cancer. For example, the role of neoantigen editing is diminished or the copy number of previous clonal neoantigens is lost during tumor evolution. Promoter hypermethylation of genes carrying neoantigens has been identified as a potential mechanism for immune editing in lung cancer (31). However, this mechanism has not been verified in ESCC, which could be further explored and elucidated in future studies. ESCC cells may also suppress the anti-tumor immune response and facilitate tumor progression by secreting some immunosuppressive cytokines that play a vital role in enhancing immune escape in the TME, including transforming growth factor-β (TGF-β), IL-6, and IL-10, etc. Previous studies have demonstrated that the expression levels of these immunosuppressive cytokines are significantly higher in ESCC tissues and are associated with a decrease in infiltrating lymphocytes in TME and a worse prognosis (32–35).

### 2.2 Cancer-associated fibroblasts

Cancer-associated fibroblasts (CAFs), as a main stromal component in the TME, have attracted increasing attention due to their important roles in tumorigenesis and tumor progression (36). CAFs, characterized by high expression of fibroblast activation protein-α and α-smooth muscle actin, are a group of activated fibroblasts with strong proliferation, migration, secretion and synthesis capabilities (37). Generally, resident fibroblasts around tumor cells are the main source of CAFs, and tumor cells can secrete various growth factors such as TGF-β to induce fibroblasts to differentiate into CAFs (38). Moreover, CAFs can interact with tumor-infiltrating immune cells within the TME through the secretion of various cytokines, growth factors, and other effector molecules, consequently creating an immunosuppressive and pro-survival TME that enables cancer cells to escape from the surveillance of the immune system (39). CAFs can reduce the expression of NK activation receptors and cytolytic proteins *via* the secretion of TGF-β, thereby inhibiting the activation and cytotoxic effects of NKs (40). In addition, TGF-β secreted by CAFs can also reduce the secretion of granzyme and perforin from cytotoxic T lymphocytes (CTLs) and inhibit cell survival proteins to promote the death of CTLs (41). Moreover, CAFs can recruit immunosuppressive cells or induce their differentiation. For example, CAFs can recruit tumor-associated neutrophils (TANs) by secreting chemokine including CXCL1, CXCL2, and CXCL5 (42); recruit monocytes to TME and differentiate them into M2 macrophages by releasing monocyte chemotactic protein 1 (MCP-1) (43); promote the differentiation of monocytes into myeloid-derived suppressor cells (MDSCs) by secreting IL-6 and signal transducer and activator of transcription 3 (STAT3) (44); stimulate CD4^+^ T cells to differentiate into Tregs and Th2-type helper T cells (45), etc. CAFs themselves can express different ligands of ICs molecules on their cell surface, such as PD-L1 and PD-L2, to induce T cell exhaustion and deactivation (46).

It has been proved that CAFs play an important role in shaping the immunosuppressive TME of ESCC through one or more of the above mechanisms. Qiu et al. found that CAFs could significantly upregulate the expression of PD-L1 in ESCC cells (47). Indirect co-culture of human bone marrow-derived mesenchymal stem cells (MSCs) with ESCC cells demonstrated that CAF-like cells promoted the cell growth and migration of ESCC cells and peripheral blood mononuclear cell-derived macrophage-like cells (33). CAF-like cells could also induce the M2 polarization of macrophage-like cells, which actively participate in the suppression of anti-tumor immune responses (33). Cui et al. found that indoleamine 2,3-dioxygenase (IDO), as an immunosuppressive factor that can induce apoptosis of effector T and NK cells while promoting Tregs activity, is expressed in CAFs and endothelial cells in the ESCC stroma, suggesting that ESCC cells may evade immune surveillance through IDO-expressing CAFs (48). Recent research in ESCC confirmed that CAFs could induce the generation of monocytic MDSCs *via* IL-6/exosomal miR-21-activated STAT3 signaling (49). Kato et al. analyzed the intratumoral and peritumoral tissues of 149 ESCC patients using immunohistochemical analysis, and found that CAFs were negatively and positively correlated with CD8^+^ tumor-infiltrating lymphocytes (TILs) and forkhead box protein 3 (FoxP3^+^) TILs in intratumoral tissues, respectively (50). Therefore, CAFs may create an immunosuppressive TME for ESCC cells by promoting the infiltration of FoxP3^+^ TILs (Tregs) while inhibiting that of the CD8^+^ TILs. Similarly, Huang et al. found a negative correlation between WNT2^+^ CAFs and active CD8^+^ T cells in primary ESCC, and anti-WNT2 monoclonal antibody (mAb) could restore anti-tumor immune responses and enhance the efficacy of anti-PD-1 in both mouse ESCC and colorectal cancer syngeneic tumor models (51). Additionally, previous studies have revealed that FGF2 overexpression in CAFs promotes sprout RTK signaling antagonist 1 (SPRY1) expression in CD8^+^ T cells and attenuates T cell receptor (TCR)-triggered T cell activation *in vitro* and *in vivo*, which could contribute to ESCC progression (52).

Therefore, novel immunotherapy targeting CAFs would immensely benefit patients with ESCC due to the central role CAFs paly in shaping the immunosuppressive TME. Recently, fibroblast activation protein (FAP), as a common biomarker of CAFs, has been extensively studied to develop immunotherapies targeting CAFs in TME in several human malignancies including ESCC (47, 53). A variety of materials, such as DNA vaccine, oncolytic adenovirus, and nanoparticles have been designed to target FAP^+^ CAFs and some of them have already entered clinical trials (54–56). Therefore, eliminating the tumor-promoting effects of CAFs by targeting FAP^+^ CAFs is a promising approach to potentially lead to better prognosis for ESCC patients. However, CAFs-targeted immunotherapy is still some way from entering clinical practice in ESCC.

### 2.3 Tumor-associated macrophages

Infiltration of tumor-associated macrophages (TAMs) is one of the hallmarks of cancer (57). The differentiation and maturation process of macrophages can be broadly divided into two different types based on different microenvironmental stimuli: the classical activation pathway produces M1 macrophages, and the bypass activation pathway produces M2 macrophages (58). M1 macrophages are believed to participate in anti-tumor immune responses, while M2 macrophages mainly play a pro-tumor role and constitute the predominant class of TAMs (59). M2-like TAMs are closely associated with the formation of immunosuppressive TME by expressing various surface markers and secreting cytokines, however, the underlying mechanisms remain largely obscure. M2-like TAMs can secrete growth factors, matrix metalloproteinases (MMPs), and other cytokines that are pro-tumor as well as inhibit T and NK cells, leading to an attenuated immune response (60). M2-like TAMs can recruit CD4^+^ Th2 type cells and Treg cells by secreting chemokines C‐C motif chemokine ligand 17 (CCL17) and CCL22 (61). In addition, M2 macrophages can express IC molecules such as PD-L1 to inhibit the proliferation and activation of T lymphocytes (62).

Previous studies have identified TAMs as important participants in the formation of the immunosuppressive TME of ESCC. Zheng et al. found that M2 macrophages was enriched in TME of ESCC, which could be associated with immune evasion and tumor progression (63). A study performed by Yagi et al. showed that increased TAMs density in EC tissues related to worse survival outcomes, and TAMs could also increase PD-L1 expression in tumor cells to induce the immune escape of them (64). The C‐C motif chemokine ligand 2 (CCL2)/C‐C motif chemokine receptor 2 (CCR2) signaling pathway is widely believed to play an important role in recruiting TAMs, subsequently contributing to tumor progression (65). Recent research in ESCC revealed that upregulated expression of CCL2 was associated with TAMs accumulation, and they were both good predictors for poor survival. Blocking the CCL2-CCR2 signaling pathway could significantly restore the anti-tumor efficacy of CD8^+^ T cells in TME by hindering TAMs recruitment. Moreover, M2 polarization of macrophages upregulated PD-L2 expression in TAMs, leading to immune evasion and tumor progression *via* PD-1 signaling pathway (66). The colony-stimulating factor 1 (CSF-1)/colony-stimulating factor 1 receptor (CSF-1R) signaling pathway has been proved to regulate the production, differentiation and activation of TAMs (67). Omstead et al. reported that the inhibition of CSF-1/CSF-1R signaling axis resulted in increased infiltration of CD8^+^ T cells with decreased M2 macrophage polarization in the TME of EAC rat model, and CSF-1R inhibitors could enhance the anti-tumor activity of PD-1/PD-L1 inhibitors by suppressing immune evasion (68). However, the roles and mechanisms of CSF-1/CSF-1R axis in immune escape of ESCC are still not fully elucidated, which is worth exploring in future studies. Therefore, TAMs represent a promisingly valuable therapeutic target for precise immunotherapy of ESCC due to their pivotal roles in cancer progression and immune evasion.

In recent years, TAMs have gradually become a hot target of tumor immunotherapy. With more and more in-depth understanding of the interaction between TAMs, TME and tumor cells, many anti-tumors therapy related explorations have been carried out in targeting TAMs (69). One strategy to target TAMs is to inhibit the recruitment of TAM precursors, that is, to prevent monocytes from being recruited into TME and prevent their activation into M2-like TAMs (69). There are currently several preclinical and clinical trials focused on inhibiting TAMs recruitment in TME by blocking CSF-1/CSF-1R signaling pathway and CCL2/CCR2 interaction in various human tumors (70–72). Nevertheless, immunotherapy targeting TAMs is still a certain distance from clinical practice. Moreover, immunotherapy targeting TAMs in TME has hardly been investigated in ESCC, and further studies in this field are expected to improve the prognosis of ESCC patients.

### 2.4 Tregs

Tregs are a distinct subpopulation of CD4^+^ T cells with immunosuppressive properties that are essential for maintaining immune homeostasis and self-tolerance, limiting excessive inflammation, and preventing autoimmunity (73). Tregs, which are universally characterized by CD4^+^CD25^+^Foxp3^+^CD127^low/−^, are an important component of the TME and play a vital role in the immune escape of tumors (74). Tregs exhibit their immunosuppressive function through various mechanisms. For example, Tregs can induce immunosuppression through the release of suppressive cytokines such as IL-10, TGF-β, and IL-35 (75–77). CTLA-4 expressed on the surface of Tregs is a ligand for CD80/CD86 with higher affinity than CD28, which downregulates CD80/CD86 co-stimulatory molecules on antigen-presenting cells (APCs), depriving T cells of co-stimulatory signals and thereby strongly inhibiting the maturation of APCs (78). In addition, Tregs mediate depletion and apoptosis of effector T cells by competitive binding of IL-2 and producing granzyme B, thereby reducing the number of effector T cells and inhibiting the immune responses (79).

Increased accumulation of Tregs has been verified both in the peripheral blood and esophageal mucosa of esophageal cancer patients, indicating that Tregs may play important roles in shaping the immunosuppressive TME of ESCC (63, 80). The chemokines CCL17 and CCL22, which are secreted by tumor cells and macrophages, have been reported as key factors in the recruitment of Tregs *via* the CCR4 receptor in ESCC (81). Previous studies have demonstrated that the increased infiltration of Tregs is associated with deep tumor invasion and worse prognosis for ESCC patients (82). Yue et al. found that IL-33 could promote the expression of CCL2 through the nuclear factor-κB (NF-κB) pathway, thereby recruiting Tregs to promote ESCC progression (83). Zhao et al. reported that elevated L1 cell adhesion molecule (L1CAM) expression in ESCC cells could facilitate CCL22 expression by activating the PI3K/Akt/NF-κB signaling pathway, thereby promoting Tregs recruitment to the tumor site (84). A recent study performed by Han et al. showed that IL-32 may have a contradictory role in the TME of ESCC, which enhanced the anti-tumor activity by promoting IFN-γ expression in CD8^+^ T cells, while suppressing the immune responses by inducing Foxp3 expression in CD4^+^ T cells (85). However, the specific mechanisms of Tregs in mediating immune evasion from ESCC are far from well-elucidated due to their heterogeneity and context-dependent functions, which is a current challenge for this field.

### 2.5 Myeloid-derived suppressor cells

MDSCs are defined as a heterogeneous group of immature myeloid cells associated with poor prognosis and survival in cancer patients. MDSCs are usually generated in the bone marrow, and they can migrate into peripheral lymphoid tissues and TME when cancer occurs, contributing to TAMs formation (86). MDSCs can suppress anti-tumor immune responses by various mechanisms. Activated MDSCs can directly inhibit NK and effector T cells *via* the production of Arginase-1 and inducible nitric oxide synthase-2 (iNOS) (87). MDSCs participate in the formation of immunosuppressive TME by secreting some immunosuppressive molecules, such as TGF-β, IL-10 and IDO (88). Moreover, MDSCs also have the ability to induce the expansion of Tregs with immunosuppressive properties (89).

Accumulated evidence has proved that MDSCs may serve as one of the most important immunosuppressive cells in TME of ESCC, which is associated with poor prognosis (90). Huang et al. found that increased circulating MDSCs were accompanied by high PD-L1 expression, indicating that MDSCs might exert immunosuppressive function *via* PD-1/PD-L1 pathway (91). It has been reported that IL-6 played crucial roles in MDSCs recruitment, and IL-6-stimulated MDSCs expressed significantly higher levels of reactive oxygen species (ROS) and arginase-1 (92). Karakasheva et al. found that MDSCs expressing high level of CD38 showed greater immunosuppressive capacity *in vivo*, and the CD38 expression was mediated by factors including IL-6, interferon-γ (IFN-γ), CXCL16, TNF-α, and IGFBP-3. Meanwhile, the number of CD38^+^ MDSCs in the peripheral blood of advanced stage EC patients was also increased (93). A study performed by Li et al. revealed that Maelstrom (MAEL) could promote the recruitment of polymorphonuclear myeloid-derived suppressor cells (PMN-MDSCs) to tumor sites *via* activation of Akt1/RelA/IL-8 signaling pathway, and TGF-β secreted by PMN-MDSCs could upregulate MAEL by inducing Smad2/Smad3 phosphorylation to further promote ESCC progression (94). Yue et al. recently reported that NEDD9 could maintain the stem-like phenotype of ESCC cells by recruiting MDSCs *via* CXCL8, suggesting NEDD9 as a novel therapeutic target for ESCC (95). Currently, the roles and mechanisms of MDSCs in the formation of immunosuppressive TME in ESCC remains obscure, and further investigation into MDSCs biology will provide direction for therapeutic approaches targeting MDSCs.

### 2.6 Tumor-associated neutrophils

Neutrophils are one of the most important inflammatory immune cells, and they are closely associated with the development of tumors (96). A large number of neutrophils are often gathered in the TME, which is defined as tumor-associated neutrophils (TANs). Similar to M1/M2 TAMs, TANs in the TME can be divided into two phenotypes: N1 and N2. Among them, N1 phenotype has the effect of inhibiting the progression of tumors by direct cytotoxicity, antibody-dependent cell-mediated cytotoxicity (ADCC) and activation of other immune cells. In contrast, N2 type TANs promote the proliferation and metastasis of tumor cells, inhibit anti-tumor immunity, and ultimately promote tumor progression (97). TGF-β within the TME can induce the polarization of TANs from N1 to N2 phenotype, thereby promoting tumor progression (98).

The important role of TANs in the development of ESCC is gradually being recognized. Recent studies revealed that higher neutrophil to lymphocyte ratio (NLR) was associated with deeper tumor invasion, lymph node metastasis, advanced TNM stage, and worse survival outcomes in ESCC patients undergoing esophagectomy (99, 100). Although the NLR is usually calculated from routine blood tests, it can still reflect changes of inflammation status in the TME (101). Neutrophil extracellular traps (NETs) is a meshwork of deoxyribonucleic acid, histones and antibacterial proteins released by activated neutrophils, which participate in the immune responses by trapping pathogens (102). However, in recent years, it has been found that NETs exist in the TME of a variety of tumors, and NETs can promote the spread and metastasis of tumor by trapping circulating tumor cells (103). Zhang et al. has demonstrated that elevated level of intra-tumoral NETs infiltration was associated with worse prognosis in both ESCC and EAC patients (104). Nevertheless, a prior study showed that IL-17 could induce accumulation and activation of myeloperoxidase^+^ TANs by stimulating ESCC cells to secret CXCL2 and CXCL3, and IL-17 could also strengthen the killing capacity of TANs by releasing various cytotoxic molecules, which predicted a favorable prognosis in ESCC patients (105). Therefore, the role of TANs in ESCC remains poorly elucidated and needs to be further investigated. Given that TANs play both tumor-suppressive and tumor-promoting roles in the process of tumor development, the full and rational utilization of their dual roles may provide new directions for ESCC immunotherapy strategy.

The role of TANs in tumor immunity has become a hot topic in recent years. How to make good use of the two sides of TANs will become a serious challenge in the future (106). Although there are various TANs-targeted therapies, little significant progress has been achieved. Up to now, several preclinical and clinical studies have evaluated the efficacy and safety of TANs-targeted therapies. Inhibition of neutrophil recruitment and polarization has been evaluated for tumor therapeutic efficacy (107–109). As for ESCC, the current challenge is to explore the role and specific mechanism of TANs in tumor immunity, so as to provide direction and reference for immunotherapy targeting TANs in TME.

### 2.7 Th17 cells

T helper cell 17 (Th17) is a newly identified subset of CD4^+^ helper T cells that secretes IL-17, which is important in autoimmune diseases and the body’s immune response (110). Although Th17 cells are prevalent in the TME, their role in tumor immunity is still controversial. Previous studies demonstrated that IL-17A expressed by Th17 cells induced ESCC cells to produce chemokines that can aggregate various immune cells such as effector T cells, B cells, DCs, and NK cells migrate to ESCC tissue and then exert their anti-tumor effects (111, 112). However, Chen et al. found that the proportion of Th17 cells increased within the peripheral blood and tumor tissues of ESCC patients, and the elevated levels of Th17 cells was positively correlated with lymph node metastasis and advanced stages (113). In addition, it has been reported that Th17 cells possess the ability to convert into Tregs, suggesting Th17 cells may participate in the immune evasion of ESCC (114). Up to now, the functions of Th17 cells in ESCC are still not well-defined, thereby understanding the role of Th17 cells in tumor immunity is crucial for the development of novel immunotherapy strategies for ESCC.

## 3 Non-cellular components

### 3.1 Cytokines

Cytokines are a heterogeneous group of small soluble peptides or glycoproteins with pleiotropic effects that promote the growth, differentiation and activation of normal cells (115). They have pro- or anti-inflammatory activity and can also produce immunosuppressive activity, depending mainly on the microenvironment surrounding the tumor. Different cells produce different cytokines in the local tumor environment, thereby regulating different biological functions of various cells (116). Previous studies have revealed that cytokines in the TME participate in the immune escape of ESCC.

TGF-β is a multifunctional cytokine that regulates cell proliferation, differentiation, adhesion, migration and apoptosis, which is one of the major players in regulating the composition and function of the TME (117). TGF-β is mainly derived from tumor cells and regulates the growth and metastasis of tumor cells in an autocrine or paracrine manner. In addition, tumor-infiltrating stromal cells, including fibroblasts, leukocytes, macrophages, bone marrow-derived endothelial cells, and mesenchymal cells, are another major source of this cytokine (118). In early-stage tumors, TGF-β exerts anti-tumor effects by promoting cell cycle arrest and apoptosis, while in advanced-stage tumors TGF-β promotes tumor metastasis and recurrence by promoting immunosuppression, promoting tumor angiogenesis, and inducing epithelial-mesenchymal transition (EMT) (119). TGF-β can directly activate Tregs to inhibit the cytotoxicity of CTLs and NK cell as well as the antigen-presenting function of APCs (120). And TGF-β can stimulate the transformation of fibroblasts into CAFs, thereby promoting the immune escape of tumor cells (121). Importantly, TGF-β can also induce macrophages to polarize toward M2 type and while activating Tregs, resulting in immunosuppressive responses (122). Blum et al. found that the TGF-β signaling axis was hyperactivated in EC patients, and the genes regulated by these pathways were also overexpressed (123). And Zhang et al. revealed that the expression of TGF-β in CAFs was significantly associated with the prognosis of ESCC patients treated with chemoradiotherapy (32). Moreover, it has been reported that CAFs could induce chemoresistance by a FOXO1/TGF-β1 signaling loop in ESCC, indicating that TGF-β may be involved in the interaction between ESCC cells and CAFs (32). Gholamin et al. found that the mRNA of TGF-β, IL-10, and VEGF were overexpressed in ESCC patients, and TGF-β was significantly co-expressed with IL-10 and vascular endothelial growth factor (VEGF), which all plays important roles in immune suppression (124). A prior study performed by Li et al. showed that esophageal cancer-derived microvesicles could induce regulatory B cells with the ability to suppress the proliferation of CD8^+^ T cells (125). Notably, previous research in ESCC demonstrated that MDSCs derived-TGF-β induced elevated PD-1 expression on CD8^+^ T cells, resulting in resistance to PD-1/PD-L1 blockade in TME. And simultaneous blockade of PD-1/PD-L1 and TGF-β signaling collaboratively restored the anti-tumor ability of CD8^+^ T cells in both vitro and vivo (126). Encouraging data from a phase I clinical study (NCT02517398) demonstrate the potential safety and clinical antitumor activity of M7824 (anti-PD-L1 and anti-TGF-β dual antibody) in multiple types of difficult-to-treat cancers, including advanced non-small-cell lung cancer (NSCLC), human papillomavirus (HPV)-associated cancers, biliary tract cancer (BTC) and gastric cancer (127). In addition, in preclinical studies, M7824 also showed stronger antitumor activity compared to anti-PD-L1 and anti-TGF-β monotherapies (128). However, Merck KGaA announced in 2021 that M7824 as the first-line treatment for NSCLC and BTC was unlikely to achieve the desired outcomes and therefore terminated three related clinical studies (NCT03631706, NCT03833661, and NCT04066491). The reason for the failure of these clinical trials may be that TGF-β is a clinically unproven target compared to PD-1/PD-L1 and CTLA-4, and the effectiveness of the clinical combination is not yet clear. Another PD-L1 and TGF-β dual antibody, SHR-1701, is in various stages of clinical trials for a variety of malignancies, and their results are promising and may provide a new direction for immunotherapy in ESCC.

Interleukin (IL) is a class of cytokine produced by lymphocytes, monocyte-macrophages and other non-mononuclear cells, which can act on a variety of cells. They play an important role in transmitting information, activating and regulating immune cells, mediating inflammatory response (129). Notably, the expression of different ILs in the TME is unbalanced. IL-4, IL-6 and IL-10 are abundantly expressed in TME and play more pro-tumor role, while IL-2, IL-12, IL-15 and IL-18, which are immunomodulatory or pro-inflammatory factors, are restricted in the TME and play more anti-tumor role (129). ILs plays a pivotal role in the progression and immune evasion of ESCC. IL-6 activates downstream molecules such as STAT3 by binding to its receptor, which allows tumor cells to survive in a highly toxic inflammatory environment (130). It has been proved that the expression of IL-6 and STAT3 in the tissues of patients with ESCC is increased and correlated, and IL-6 and STAT3 are both independent poor prognostic factors of ESCC (131). Additionally, increased levels of CAFs-derived IL-6 promoted the migration of ESCC cells, and IL-6 was associated with the formation of immunosuppressive TME (33). Moreover, Chen et al. found that silencing B7-H4 significantly reduced IL-6 secretion, inhibited STAT3 activation, and reduced the amount of p-STAT3 entering the nucleus from the cytoplasm, thereby inhibiting the proliferation of ESCC cells (132). Wang et al. revealed that the level of IL-10 in the serum of ESCC patients was significantly increased, and the IL-10 level was positively associated with Tregs density (35). In addition, a recent study has demonstrated that IL-32 enhanced the anti-tumor activity by promoting IFN-γ expression in CD8^+^ T cells, while suppressing the immune responses by inducing Foxp3^+^ Tregs cells, indicating its contradictory role in the TME of ESCC (85). Currently, immunotherapeutic drugs designed for ILs are mainly agonists, including IL-2, IL-12, IL-15, etc (133). However, there are still limitations in clinical application of these agents, mainly because of the poor specificity of cytokine action and the high risk of immune-related adverse events (irAE). To solve this problem, some manufacturers now couple the cytokine agonist with an antibody (such as PD-1 antibody), thus giving some specificity to this cytokine agonist. In conclusion, ILs are key elements in the coordination of TME and tumor-immune cell interactions, and interleukin-related immunotherapy for various cancer has led to a wealth of exciting new developments in preclinical and clinical studies that will yield more interesting results in the coming years.

Chemokines are a class of small cytokines or signaling proteins secreted by various cells that have the ability to induce directional chemotaxis of nearby responding cells (134). Based on the location of the first two cysteine (C) residues of their primary protein structure, chemokines are generally divided into four subclasses: C, CC, CXC and CX_3_C chemokines. Chemokines participate in the formation of immunosuppressive microenvironment by recruiting immunosuppressive cells such as Tregs, MDSCs and TAMs to the TME, indicating that they play an important role in the immune evasion of ESCC (135). As mentioned above, the chemokines CCL2, CCL17 and CCL22 have been reported as key factors in the recruitment of Tregs *via* their corresponding receptor in ESCC (81, 83, 84). In addition, Liu et al. has revealed that chemokine CCL20 was positively correlated with Treg markers in ESCC tissues, and CCL20 could recruit Tregs *in vitro* by binding to its receptor CCR6 (136). Recent research in ESCC revealed that upregulated expression of CCL2 could mediate TAMs accumulation *via* the CCL2-CCR2 signaling pathway, thereby inhibiting the anti-tumor ability of CTLs in TME and promoting immune evasion of ESCC cells (66). Moreover, Yue et al. demonstrated that CXCL8 mediated the recruitment of MDSCs induced by NEDD9 *in vitro* and *in vivo*, which promoted the stemness of ESCC cells (95). A previous study conducted by Chen et al. showed that the CCL17/22- CCR4 and CCL20-CCR6 axes might be of considerable significance in Th17 cells infiltration in TME of ESCC (137). Given the important roles chemokines play in the aggregation of immunosuppressive cells in TME, chemokines hold promise as potential therapeutic targets for ESCC patients. At present, chemokine-based immunotherapies include two main types: targeting pro-tumor chemokines and increasing the concentration of anti-tumor chemokines. Both can be used either as stand-alone therapies or in combination with other therapeutic strategies (138). Carlumab, a high-affinity monoclonal antibody that specifically targets CCL2, inhibits CCL2-mediated migration of TAMs to tumors. Despite showing robust therapeutic effects in preclinical mice models, treatment with carlumab failed to affect response rates in some clinical trials (NCT00537368 and NCT01204996) and was therefore discontinued (138, 139). HuMax-IL8 is an antibody approach specifically targeting CXCL8. Preclinical studies have shown that blocking CXCL8 with HuMax-IL8 reduces the recruitment of MDSCs to TME (140). Results from the phase I clinical trial demonstrated that serum CXCL8 levels were reduced, although no objective tumor response was detected (141). Together, immunotherapies that target chemokines have promising potential and feasibility, although their clinical potential remains to be demonstrated.

### 3.2 Hypoxia, acidosis, and metabolism

Hypoxia and low pH are characteristic manifestations in the TME, which are closely associated with a series of metabolic changes in tumors (142). For solid tumors, the rapid proliferation and energy metabolism of tumor cells in a relatively confined space with delayed vascular development and poor blood perfusion make hypoxia the norm in the TME (143). The hypoxic microenvironment can activate signaling pathways such as hypoxia-inducible factor 1 (HIF-1) in tumor cells to alter the biological behavior and material-energy metabolism to adapt to the hypoxic environment (144, 145). In turn, tumor cells can modify the TME by releasing biological molecules such as MMP, VEGF and metabolites such as lactic acid, changing the ECM, increasing angiogenesis and changing the pH value of the microenvironment, which have an impact on drug delivery, hemoperfusion and immunological status in the TME (146–148). To a certain extent, it can also enhance the tolerance of tumors to radiotherapy, chemotherapy and immunotherapy (149, 150). It has been proven that hypoxia can lead to the upregulation of some cytokines in the TME such as NF-κB, IL-6, IL-8, TGF-β, and IL-10 (151, 152). Hypoxic TME can promote the recruitment of immunosuppressive cells including Tregs, TAMs and MDSCs (153). Additionally, nutrient deficiencies and accumulation of metabolites in the TME pose a great challenge for immune cells to exert their anti-tumor effects, but cancer cells themselves are not affected by microenvironment selection (154). In recent years, the role of hypoxia in the immune escape of ESCC has gradually attracted attention. Previous research on ESCC has showed that HIF-1α expression was significantly associated with venous invasion, VEGF expression, and microvessel density, and ESCC patients with high HIF-1α-expressing tumors had worse survival outcomes (155). Lu et al. revealed that hsa-circ-0048117-rich exosomes generated by ESCC cells in a hypoxic TME could promote M2 macrophage polarization and favors the malignant behaviors of ESCC cells (156). A recent study has identified six hypoxia-related lncRNAs (HRlncRNAs) that were associated with the prognosis of digestive system pan-cancer, and the HRlncRNA high expression group was positively correlated with increased tumor-infiltrating immune cells such as CAFs, macrophages, and myeloid dendritic cell, while they were negatively correlated with CD4^+^ T cells, suggesting an immunosuppressive TME (157). However, the role of hypoxia in the formation of the immunosuppressive TME of ESCC and its specific mechanisms are still not well elucidated, which provides a direction for future research.

It is well recognized that hypoxic TME is usually accompanied by an increase in lactate concentration due to the “Warburg effect”. The Warburg effect means that even with sufficient oxygen, tumor cells obtain energy predominantly through the glycolysis pathway rather than the tricarboxylic acid (TCA) cycle, resulting in lactate accumulation in the TME. Lactate accumulation in TME can induce polarization of M2-type TAMs and N2-type TANs (158, 159). In addition, acidic TME induced by lactate produced by tumor cells can inhibit the anti-tumor immune responses of CTLs and NK cells (160, 161). It has been proved that the activity and recruitment of Tregs were increased in acidic TME, and the expression of Foxp3 in Tregs is also significantly increased, which enhanced the immunosuppressive effect of Tregs (73, 162). Additionally, excessive amounts of lactic acid in TME increased PD-1 expression and suppressive activity of Treg cells. PD-1 blockade could also enhance immunosuppressive activities of effector Treg cells in a high-lactate environment, and the efficacy of PD-1 blockade therapy was recovered by targeting lactic acid metabolism of Treg cells, indicating that lactic acid is a factor in immunotherapy resistance (163). Acidosis also plays an important role in the immune escape of EC. Carbonic Anhydrase IX (CAIX), as a target gene of HIF-1α, acts as a key molecule in maintaining the pH value of cells in a hypoxic microenvironment. Previous study has demonstrated that the expression of CAIX increased in EC, and CAIX could promote the migration and metastasis of EC cells while maintaining intracellular pH stability (164). Moreover, elevated level of CAIX expression was associated with greater aggressiveness and worse survival outcomes of ESCC (165). In hypoxic TME, a series of metabolites produced by tumor cells and inflammatory cells, including lactic acid, ROS, IDO, prostaglandin E2 (PGE2), soluble fatty acids and adenosine and other molecules, can accelerate the aging and exhaustion of immune cells, creating a zone of immunosuppression (166, 167). In recent years, the roles of hypoxia, acidosis and metabolism in the formation of immunosuppressive TME have gradually attracted attention, which is expected to provide a new target for the immunotherapy of ESCC.

## 4 ICIs drug resistance and TME

Over the past decade, ICIs has been successfully applied in the clinical practice as a novel therapy for treating ESCC, bringing new hope to ESCC patients. However, the overall response rate in patients with ESCC is lower than 30%, and the majority of patients initially treated with ICIs are likely to develop acquired resistance over time (120). The efficiency and durability of ICIs were greatly weakened by immunosuppression. Notably, the mechanisms of drug resistance is a complex multifactor process, including blockage of drug distribution, mutations of the drug target, increased drug efflux, and evasion of programmed cell death (168). Development of drug resistance is not only related to malignant hallmarks of tumor cells, but also closely associated with the immunosuppressive TME and the crosstalk between tumor cells and TME (150). It has been widely proved that TME plays an important role in the therapeutic response of various human cancer (169). However, the exact mechanism of ICIs resistance in ESCC remains elusive due to the heterogeneity within the immune TME and the highly diverse oncological characteristics in different ESCC individuals.

The presence of multiple ICs (including PD-1/PD-L1, CTLA-4, TIGIT, TIM, and LAG-3, etc.) is one reason for the development of drug resistance. Current immunotherapies generally block only one or two immune checkpoints, and unblocked immune checkpoints can still exert immunosuppressive effects. Some studies have found that many patients treated with chemotherapy plus immunotherapy develop primary or secondary drug resistance due to the existence of ICs such as TIGIT and TIM (170, 171). Several ongoing clinical trials (NCT04732494, NCT04140500, NCT03708328) combining anti-PD-1 and anti-TIGIT/TIM/LAG-3 treatment as novel immunotherapies are expected to provide better prognosis for ESCC patients. In addition to inhibitory molecules expression, ESCC cells could develop drug resistance by secreting immunosuppressive cytokines and growth factors (120). Previous studies have demonstrated that T cells activated by ICIs therapy preferentially recognize mutant antigens (172). The activation process of T cells is largely dependent on the MHC molecules of APCs, and tumor cell antigens presented through MHC class I molecules are regulated by a variety of genes. Thus, these neoantigens presented by APCs are also downregulated when genetic deletions, epigenetic alterations, or mutations, which may lead to acquired resistance of ICIs therapy. Beta-2-microglobulin (B2M) is a crucial gene involved in stabilizing the MHC class I molecules at the cell surface. Gettinger et al. found that B2M knockout cells were less sensitive to PD-1 blockade in lung cancer, indicating that B2M loss might confer drug resistance to ICIs therapy (173). Genetic mutations occur frequently during tumor growth, which generates neoantigens and affects the response to ICIs therapy (174). Patients with rich expression of neoantigens are more sensitive to ICIs treatment and obtain more clinical benefits, while loss of neoantigen mutations may lead to immune evasion and resistance to ICIs therapy (31, 175). Moreover, the above-mentioned immunosuppressive cells, including CAFs, TAMs, MDSCs, and Tregs may also participate in the development of drug resistance in ESCC patients through multiple pathways. Further exploration of the drug resistance mechanisms of immunotherapy is helpful for the development of new drugs, and provides ideas for further research and guidance for clinical treatment. Current studies have revealed some resistance mechanisms of immunotherapy in ESCC, but there are still more problems that need to be solved. With the continuous progress of molecular biology and gene sequencing technology, more drug resistance mechanisms will be discovered in the future.

## 5 Clinical trials on immunotherapy targeting the TME of ESCC

Tumor immunotherapy brings new hope to patients with cancers including ESCC, and in recent years immunotherapy has made breakthroughs in the field of basic research and clinical treatment. Several clinical trials have highlighted TME as a potential therapeutic target for ESCC, and some ongoing clinical trials are expected to provide broader prospects for the immunotherapy of ESCC. At present, immunotherapy strategies mainly focus on the combination of immunotherapy with chemotherapy or radiotherapy and dual immune blockade. We have summarized the available clinical trials on immunotherapy targeting the TME of ESCC registered with clinicaltrials.gov and presented them in **Table 1**.

**Table 1:** Overview of clinical trials with immunotherapies targeting immunosuppressive TME in ESCC.

Identifier	Drug/Treatment	Target	Ethnicity	Phase	Status	Result	Ref.
NCT03189719 (KEYNOTE-590)	Pembrolizumab + CT *vs.* Placebo + CT (first-line)	PD-1	Asian and Western	III	Active, not recruiting	Available	(8)
NCT03143153 (CheckMate-648)	Nivolumab + Ipilimumab *vs.* Nivolumab + CT *vs.* CT (first-line)	PD-1; CTLA-4	Asian and Western	III	Active, not recruiting	Available	(176)
NCT03691090 (ESCORT-1st)	Camrelizumab + CT *vs.* Placebo + CT (first-line)	PD-1	Asian	III	Completed	Available	(177)
NCT03829969 (JUPITER-06)	Toripalimab + CT *vs.* Placebo + CT (first-line)	PD-1	Asian	III	Active, not recruiting	Available	(178)
NCT03748134 (ORIENT-15)	Sintilimab + CT *vs.* Placebo + CT (first-line)	PD-1	Asian and Western	III	Recruiting	Available	(179)
NCT03469557	Tislelizumab + CT (first-line)	PD-1	Asian	II	Completed	Available	(180)
NCT02564263 (KEYNOTE-181)	Pembrolizumab *vs.* CT (second-line)	PD-1	Asian and Western	III	Completed	Available	(9)
NCT02569242 (ATTRACTION-3)	Nivolumab *vs.* CT (second-line)	PD-1	Asian and Western	III	Completed	Available	(181)
NCT03099382 (ESCORT)	Camrelizumab *vs.* CT (second-line)	PD-1	Asian	III	Completed	Available	(182)
NCT03430843 (RATIONALE-302)	Tislelizumab *vs.* CT (second-line)	PD-1	Asian and Western	III	Active, not recruiting	Available	(183)
NCT01938612	Durvalumab monotherapy/Durvalumab + tremelimumab (≥ second-line)	PD-L1; CTLA-4	Asian	I	Completed	Available	(184)
NCT02054806 (KEYNOTE-028)	Pembrolizumab (≥ third-line)	PD-1	Asian and Western	Ib	Completed	Available	(185)
NCT02559687 (KEYNOTE-180)	Pembrolizumab (≥ third-line)	PD-1	Asian and Western	II	Completed	Available	(186)
NCT03792347 (PALACE-1)	Preoperative pembrolizumab + CRT (neoadjuvant therapy)	PD-1	Asian	Ib	Completed	Available	(187)
NCT02743494 (CheckMate-577)	Nivolumab *vs.* Placebo after surgery (adjuvant therapy)	PD-1	Asian and Western	III	Active, not recruiting	Available	(188)
NCT04210115 (KEYNOTE-975)	Pembrolizumab + dCRT *vs.* placebo + dCRT	PD-1	Asian and Western	III	Recruiting	Not available	N/A
NCT04435197 (PALACE-2)	Preoperative pembrolizumab + CRT	PD-1	Asian	II	Recruiting	Not available	N/A
NCT04389177 (KEYSTONE-001)	Neoadjuvant pembrolizumab + nCT followed by surgery	PD-1	Asian	II	Recruiting	Not available	N/A
NCT04807673 (KEYSTONE-002)	Neoadjuvant pembrolizumab + nCT + surgery vs. nCRT + surgery	PD-1	Asian	III	Recruiting	Not available	N/A
NCT05302011	Neoadjuvant pembrolizumab + nCT	PD-1	Asian	II	Recruiting	Not available	N/A
NCT05130684	Neoadjuvant nivolumab + nCRT	PD-1	Asian	II	Recruiting	Not available	N/A
NCT03914443 (FRONTiER)	Neoadjuvant nivolumab + nCT	PD-1	Asian	I	Active, not recruiting	Not available	N/A
NCT05213312	Neoadjuvant nivolumab + nCT *vs.* nCT	PD-1	Asian	II/III	Recruiting	Not available	N/A
NCT03416244 (RAMONA)	Nivolumab + Ipilimumab *vs.* Nivolumab	PD-1; CTLA-4	Western	II	Completed	Not available	N/A
NCT04426955	Camrelizumab + dCRT *vs.* Placebo + dCRT	PD-1	Asian	III	Active, not recruiting	Not available	N/A
NCT05050760	Camrelizumab + CT	PD-1	Asian	N/A	Recruiting	Not available	N/A
NCT04741490	Postoperative camrelizumab + RT	PD-1	Asian	N/A	Recruiting	Not available	N/A
NCT04286958	Camrelizumab after dCRT	PD-1	Asian	II	Recruiting	Not available	N/A
NCT04767295; NCT05476380	Neoadjuvant camrelizumab + nCT	PD-1	Asian	II	Recruiting	Not available	N/A
NCT04506138	Neoadjuvant camrelizumab + nCT	PD-1	Asian	I/II	Active, not recruiting	Not available	N/A
NCT04225364	Neoadjuvant camrelizumab + nCT	PD-1	Asian	II	Completed	Not available	N/A
NCT05176002	Neoadjuvant camrelizumab + nRT	PD-1	Asian	II	Recruiting	Not available	N/A
NCT05507411 (WATCHER)	Neoadjuvant camrelizumab + cCRT	PD-1	Asian	II	Recruiting	Not available	N/A
NCT04005170	Toripalimab + dCRT	PD-1	Asian	II	Completed	Not available	N/A
NCT04848753	Neoadjuvant toripalimab + nCT *vs.* Placebo + nCT	PD-1	Asian	III	Recruiting	Not available	N/A
NCT04280822	Neoadjuvant toripalimab + nCT *vs.* nCT	PD-1	Asian	III	Recruiting	Not available	N/A
NCT04644250; NCT05424432	Neoadjuvant toripalimab + nCRT	PD-1	Asian	II	Recruiting	Not available	N/A
NCT04844385	Neoadjuvant toripalimab + nCT + cCRT	PD-1	Asian	II	Recruiting	Not available	N/A
NCT04177797	Neoadjuvant toripalimab + nCT	PD-1	Asian	II	Active, not recruiting	Not available	N/A
NCT04804696	Neoadjuvant toripalimab + nCT	PD-1	Asian	II	Recruiting	Not available	N/A
NCT03783442	Tislelizumab + CT	PD-1	Asian	III	Active, not recruiting	Not available	N/A
NCT03957590 (RATIONALE-311)	Tislelizumab + dCRT vs. Placebo + dCRT	PD-1	Asian	III	Active, not recruiting	Not available	N/A
NCT05394415 (LATE)	Tislelizumab + CRT for conversion therapy	PD-1	Asian	I/II	Recruiting	Not available	N/A
NCT05520619 (EC-CRT-002)	Tislelizumab + dCRT with maintenance *vs.* Tislelizumab + dCRT without maintenance	PD-1	Asian	II	Recruiting	Not available	N/A
NCT04973306 (iCROSS)	Neoadjuvant tislelizumab +nCRT *vs.* nCRT	PD-1	Asian	II/III	Recruiting	Not available	N/A
NCT05323890; NCT04776590	Neoadjuvant tislelizumab + nCRT	PD-1	Asian	II	Recruiting	Not available	N/A
NCT04732494 (AdvanTIG-203)	Tislelizumab + Ociperlimab *vs.* Tislelizumab + Placebo	PD-1; TIGIT	Asian and Western	II	Recruiting	Not available	N/A
NCT05495152	Postoperative sintilimab *vs.* postoperative follow-up	PD-1	Asian	III	Active, not recruiting	Not available	N/A
NCT04212598	Sintilimab after dCRT	PD-1	Asian	II	Recruiting	Not available	N/A
NCT03985046	Sintilimab + CT after dCRT	PD-1	Asian	II	Active, not recruiting	Not available	N/A
NCT05174325; NCT04548440	Neoadjuvant sintilimab + nCT	PD-1	Asian	II	Recruiting	Not available	N/A
NCT03946969	Neoadjuvant sintilimab + nCT	PD-1	Asian	II	Active, not recruiting	Not available	N/A
NCT05357846	Neoadjuvant sintilimab + nCRT *vs.* nCRT	PD-1	Asian	III	Not yet recruiting	Not available	N/A
NCT04543617 (SKYSCRAPER-07)	Atezolizumab + tiragolumab *vs.* Atezolizumab + tiragolumab placebo *vs.* Atezolizumab placebo + tiragolumab placebo	PD-L1; TIGIT	Asian and Western	III	Recruiting	Not available	N/A
NCT04540211 (SKYSCRAPER-08)	Atezolizumab + Tiragolumab + CT *vs.* Placebo + CT	PD-L1; TIGIT	Asian	III	Active, not recruiting	Not available	N/A
NCT04550260 (KUNLUN)	Durvalumab + dCRT *vs.* Placebo + dCRT	PD-L1	Asian and Western	III	Recruiting	Not available	N/A
NCT02520453	Durvalumab after surgery *vs.* Placebo after surgery	PD-L1	Asian	II	Active, not recruiting	Not available	N/A
NCT04851132	Durvalumab + RT	PD-L1	Asian	N/A	Recruiting	Not available	N/A
NCT04568200	Neoadjuvant durvalumab + nCRT *vs.* Placebo + nCRT	PD-L1	Asian	II	Recruiting	Not available	N/A
NCT03377400	Durvalumab + tremelimumab + dCRT	PD-L1; CTLA-4	Asian	II	Active, not recruiting	Not available	N/A
NCT02658214	Durvalumab + tremelimumab + CT	PD-L1; CTLA-4	Asian	I	Completed	Not available	N/A
NCT03212469 (ABIMMUNE)	Durvalumab + tremelimumab + SBRT	PD-L1; CTLA-4	Western	I/II	Recruiting	Not available	N/A
NCT04140500	RO7247669	PD-1; LAG-3	Asian and Western	I	Recruiting	Not available	N/A
NCT03708328	RO7121661	PD-1; TIM-3	Asian and Western	I	Active, not recruiting	Not available	N/A
NCT04785820	RO7247669/RO7121661 *vs.* Nivolumab	PD-1; LAG-3; TIM-3	Asian and Western	II	Recruiting	Not available	N/A

CT, chemotherapy; nCT, neoadjuvant chemotherapy; RT, radiotherapy; nRT, neoadjuvant radiotherapy; CRT, chemoradiotherapy; nCRT, neoadjuvant chemoradiotherapy; dCRT, definitive chemoradiotherapy; cCRT, concurrent chemoradiotherapy; SBRT, stereotactic body radiation therapy; PD-1, programmed cell death protein 1; PD-L1, programmed cell death ligand 1; CTLA-4, cytotoxic T lymphocyte-associated antigen 4; TIGIT, T-cell immunoglobulin and immunoreceptor tyrosine-based inhibitory motif domain; LAG-3, lymphocyte activation gene-3; TIM-3, T-cell immunoglobulin and mucin domain-containing protein-3; Ref., reference.

Currently, immunotherapy for ESCC is still focused on ICIs. Over the past decade, numerous clinical studies have shown that ICIs can increase remission rates and improve prognosis of ESCC patients. PD-1/PD-L1 and CTLA-4 represent the two major targetable ICs. In the field of tumor immunotherapy, PD-1/PD-L1 have attracted much attention and have been approved by the Food and Drug Administration (FDA) for the treatment of a variety of cancers. The common PD-1 inhibitors currently in clinical trials include pembrolizumab, nivolumab, camrelizumab, toripalimab, tislelizumab, and sintilimab, while atezolizumab and durvalumab are major PD-L1 inhibitors. Clinical studies have shown that some PD-1/PD-L1 inhibitors can be applied to the first-line treatment of advanced ESCC. The KEYNOTE-590 study showed that pembrolizumab in combination with chemotherapy (5-fluorouracil + cisplatin) demonstrated better efficacy and safety than placebo plus chemotherapy as first-line treatment for patients with locally advanced/unresectable or metastatic ESCC [OS: median 12.4 vs 9.8 months, P < 0.0001; progression-free survival (PFS): median 6.3 vs 5.8 months, P < 0.0001] (8). The CheckMate-648 is a study of nivolumab in combination with chemotherapy or ipilimumab (anti-CTLA-4 monoclonal antibody) in the first-line treatment of ESSC, and the resulted demonstrated that the nivolumab plus chemotherapy (5-fluorouracil + cisplatin) group (median 13.2 vs 10.7 months, P = 0.002) and nivolumab plus ipilimumab group (median 12.7 vs. 10.7 months, P = 0.01) both achieved significantly prolonged OS compared with chemotherapy alone (176). The phase III clinical trial ESCORT-1st revealed that camrelizumab in combination with chemotherapy (paclitaxel + cisplatin) in first-line treatment of patients with advanced or metastatic ESCC significantly prolonged PFS and OS (PFS: median 6.9 vs 5.6 months, P < 0.001; OS: median 15.3 vs 12.0 months, P = 0.001) with an overall good safety profile (177). In addition, the JUPITER-06 study was designed to compare the efficacy and safety of toripalimab in combination with chemotherapy (paclitaxel + cisplatin) versus placebo in combination with chemotherapy in the first-line treatment of advanced or metastatic ESCC, and the results showed that OS and PFS were improved in the toripalimab group (OS: median 17 versus 11 months, P = 0.0004; PFS: median 5.7 vs 5.5 months, P < 0.0001) with a worse but manageable safety profile (178). The ORIENT-15 study is a phase III clinical trial investigating sintilimab in combination with chemotherapy (cisplatin + paclitaxel/cisplatin + 5-fluorouracil) for the first-line treatment of advanced or metastatic ESCC (179). The results showed that sintilimab plus chemotherapy was more effective than chemotherapy alone (OS: median 16.7 versus 12.5 months, P < 0.001; PFS: median 7.2 vs 5.7 months, P < 0.001), and the treatment effect was more significant in PD-L1-positive population. Moreover, the phase II study (NCT03469557) showed that first-line tislelizumab plus chemotherapy (cisplatin + fluorouracil) demonstrated durable responses with manageable tolerability in patients with locally advanced or metastatic ESCC [PFS: 10.4 months (95% CI, 5.55–15.11); duration of response (DoR): 12.8 months (95% CI, 3.5-12.8)] (180).

Clinical trials exploring the use of PD-1/PD-L1 inhibitors for second-line and beyond treatment of ESCC have also made some progress. The KEYNOTE-181 trial is the first study to use immunotherapy in second-line ESCC treatment, and the results showed that pembrolizumab prolonged OS versus chemotherapy (paclitaxel/docetaxel/irinotecan) as second-line therapy for advanced ESCC in PD-L1-positive patients (median 8.2 vs 7.1 months, P = 0.0095), with fewer treatment-related adverse events (9). The phase III clinical trial ATTRACTION-3 revealed that nivolumab was associated with a significant improvement in OS with a favorable safety profile compared with chemotherapy (paclitaxel/docetaxel) as a second-line treatment option for ESCC patients (median 10.9 vs 8.4 months, P = 0.019) (181). Moreover, the ESCORT study revealed that camrelizumab monotherapy could significantly improve PFS and OS compared with chemotherapy (docetaxel/irinotecan) in patients with advanced or metastatic ESCC who failed first-line chemotherapy with a manageable safety profile (OS: median 8.3 versus 6.2 months, P = 0.001; PFS: median 1.9 vs 1.9 months, P < 0.001) (182). The RATIONALE-302 is a phase III clinical trial comparing the efficacy of tislelizumab with chemotherapy (paclitaxel/docetaxel/irinotecan) in the second-line treatment of patients with advanced or metastatic ESCC, and the resulted demonstrated that tislelizumab significantly improved OS compared with chemotherapy (median 8.6 vs 6.3 months, P = 0.0001) with a manageable safety profile (183). The NCT01938612 is a phase I clinical trial to evaluate the safety, tolerability, and antitumor activity of durvalumab monotherapy, and durvalumab plus tremelimumab (anti-CTLA-4 monoclonal antibody) combination therapy in patients from Asia with ESCC as second-line and beyond treatment, which showed that both treatments displayed acceptable safety profiles clinical benefit (184). The results of phase Ib clinical trial KEYNOTE-028 showed that pembrolizumab demonstrated durable antitumor activity and manageable toxicity in patients with heavily pretreated, PD-L1-positive advanced ESCC [objective response rate (ORR): 30% (95% CI, 13%-53%); DoR: 15 months (range, 6-26 months); OS: 7.0 months (95% CI, 4.3-17.7 months); PFS: 1.8 months (95% CI, 1.7-2.9 months)] (185). The KEYNOTE-180 study is a phase II clinical trial that investigated and evaluated the efficacy and safety of pembrolizumab in third-line and post-third-line treatment of advanced ESCC, which revealed that pembrolizumab could provide durable anti-tumor activity with tolerable safety in patients with heavily pretreated ESCC [ORR: 14.3% (95% CI, 6.7%-25.4%); OS: 6.8 months (95% CI, 5.4-8.9 months); PFS: 2.1 months (95% CI, 2.0 - 2.4 months)] (186). Additionally, The ATTRACTION-1 study, an open, multicenter phase II study of nivolumab given to patients with advanced ESCC who had failed or were intolerant to previous treatments, confirmed the good antitumor activity and controlled safety profile of nivolumab in third-line and beyond for the treatment of advanced ESCC (ORR: 17.2%; DoR: 11.2 months; OS: median 10.8 months; PFS: median 1.5 months) (189).

Clinical trials of ICIs as neoadjuvant immunotherapy have also made some progress. The PALACE-1 is a phase Ib clinical trial aiming to investigate the safety and activity of preoperative pembrolizumab combined with chemoradiotherapy for resectable ESCC, and the results showed that it was safe, did not delay surgery, and induced a pathologic complete response (pCR) in 55.6% of resected tumors (187). However, because PALACE-1 is only a Phase Ib clinical study, the role of preoperative neoadjuvant immunotherapy in the multidisciplinary management of ESCC is still far from well elucidated. Furthermore, results from ongoing clinical trials that incorporate immunotherapy into neoadjuvant regimens in ESCC (NCT04435197, NCT04389177, NCT04807673, NCT04848753, NCT04973306, and NCT05357846 etc.) are eagerly awaited to clarify safety and efficacy and whether immunotherapy can improve response to neoadjuvant treatment, which is highly relevant for overall survival of patients with ESCC. In addition, PD-1 inhibitors also showed promise in post-operative adjuvant therapy after radical surgery. The CheckMate-577 study revealed that nivolumab could be used as postoperative maintenance therapy for EC patients treated with neoadjuvant concurrent chemoradiotherapy and R0 resection [disease-free survival (DFS): 22.4 vs 11.0 months, P < 0.001], with significant improvement in prognosis (188). However, most of the participants in this study were patients from Western countries, and an ongoing clinical trial (NCT02520453) in China to explore the role of immunotherapy in the postoperative adjuvant treatment of ESCC is expected to contribute to this field.

CTLA-4 is another well-recognized immune checkpoint, and several clinical trials has proven the effectiveness of anti-CTLA-4 monoclonal antibody as a novel immunotherapy for comprehensive treatment of ESCC. The common CTLA-4 inhibitors currently in clinical trials mainly include ipilimumab and tremelimumab, and CTLA-4 inhibitors often combined with PD-1/PD-L1 inhibitors as dual immune blockade. As mentioned above, two clinical trials, CheckMate-648 and NCT01938612, have confirmed the efficacy and safety of combined CTLA-4 and PD-1 inhibitors as the immunotherapy of ESCC (176, 184). Several ongoing clinical trials (NCT03416244, NCT03377400, NCT02658214 and NCT03212469) are eagerly awaited to further clarify safety and efficacy of CTLA-4 inhibitors in immunotherapy of ESCC.

Some newly emerging ICs, including LAG-3, TIM-3, and TIGIT, have been identified to date and their efficacy are being investigated in several ongoing clinical trials. The AdvanTIG-203 (NCT04732494), SKYSCRAPER-07 (NCT04543617), and SKYSCRAPER-08 (NCT04540211) are clinical studies to investigate the efficacy and safety of anti-TIGIT and anti-PD-1/PD-L1 combination therapy for patients with ESCC. The NCT04140500 and NCT04785820 studies aimed to explore the efficacy and safety of anti-LAG-3 and anti-PD-1 combination therapy, while the NCT03708328 and NCT04785820 are designed to investigate the efficacy and safety of anti-TIM-3 and anti-PD-1 combination therapy for patients with ESCC. These ongoing clinical trials hold the promise of more diverse immunotherapy approaches for the comprehensive treatment of ESCC.

Although significant progress has been made in ESCC immunotherapy, only a small fraction of patients can actually achieve long-term benefits. Therefore, accurate screening of the best benefit groups by potential markers is an urgent problem to be solved, which is more in line with the concept of precision therapy. PD-L1 is a common immune biomarker, and detection of PD-L1 expression level is the most widely used method in clinical research. KEYNOTE-181 study confirmed that CPS ≥ 10 can be used as a biomarker to predict the efficacy of immunotherapy for esophageal cancer (9). Microsatellite instability-high (MSI-H), mismatch repair deficiency (dMMR) and tumor mutation burden (TMB) may also be potential biomarkers for predicting the efficacy of immunotherapy in ESCC (190). Additionally, with the advance of immunotherapy to the first-line treatment of advanced ESCC, the problem of drug resistance has gradually emerged. Some studies have found that in the chemotherapy combined with immunotherapy, due to the existence of ICs such as TIGIT and TIM, many patients develop primary or secondary drug resistance (170, 171). One potential strategy to overcome the limitations of PD-1 immunotherapy is to target additional immune checkpoints associated with the immunosuppressive TME. Several ongoing clinical trials combining anti-PD-1 and anti-TIGIT/TIM/LAG-3 treatment as novel immunotherapies are expected to provide better prognosis for ESCC patients. Moreover, the immunotherapy of ESCC is mainly based on ICIs currently. With the further research on the pathogenesis of ESCC, novel immunotherapies targeting immunosuppressive TME (such as CAFs, TAMs, Tregs, MDSCs and cytokines) will be available in the near future, which is promising to create a new era for the immunotherapy of ESCC.

## 6 Conclusions and perspectives

The TME is an indispensable participant in immune evasion of ESCC. TME is a dynamic and ever-changing biological system consisting of various cellular and non-cellular components, which is responsible for immune escape to some extent. Increasing evidence has demonstrated that the formation of immunosuppressive microenvironment in ESCC is a complex process, involving not only the tumor cells themselves, but also CAFs, TAMs, Tregs, MDSCs, TANs, cytokines, hypoxia, acidosis and metabolism. At present, immunotherapies have been developed that are often used in combination with conventional chemotherapy aim to promote treatment efficacy. Although significant improvement has been reached, responses to immunotherapies and prognosis in ESCC patients are still far from satisfactory.

The current challenges are to devote considerable effort to the immunological and biological exploration of ESCC to more accurately tune the various existing or emerging immunotherapies. Although previous studies have provided clues to the important role of various immunosuppressive cells in the TME of ESCC, further studies are needed to investigate their functions in the occurrence and progression of ESCC and the formation of the immunosuppressive TME. Meanwhile, the inadequacy and imbalance of previous studies may lead to incomplete assessment of the complex immune contexture in ESCC. For example, the role and mechanism of TANs and Th17 cells in the formation of ESCC immunosuppressive TME are still controversial, which are supposed to be further clarified in future studies. Another urgent challenge is to find biomarkers that can accurately predict patients’ responsiveness and drug resistance to immunotherapy. At present, there are few biomarkers existing or applied in daily clinical practice in ESCC, and further research is urgently needed.

With the further exploration of the formation mechanism of ESCC immunosuppressive TME, there will be more immunotherapeutic methods targeting immunosuppressive TME. By targeting key molecules and immune cells in the TME, different combinations of chemotherapy, molecular targeted therapy and immunotherapy are promising to further improve the prognosis of ESCC patients. A number of ongoing preclinical and clinical studies hold great promise to maximize ESCC patients’ benefits from an immunotherapeutic approach targeting tumor-favorable TME in the foreseeable future.

## Author contributions

Conception and design: ZS and RL. Administrative support: ZS and HT. Provision of study materials or patients: RL and BH. Collection and assembly of data: RL. Data analysis and interpretation: RL. All authors contributed to the article and approved the submitted version.
